# Posterior intervertebral space debridement, annular bone grafting and instrumentation for treatment of lumbosacral tuberculosis

**DOI:** 10.1186/s12893-017-0310-2

**Published:** 2017-12-04

**Authors:** Weiwei Li, Jun Liu, Liqun Gong, Yongchun Zhou, Dapeng Duan

**Affiliations:** 1 Department of Orthopedic, Shaanxi Provincial People’s Hospital, Xi’an, Shaanxi 710068 China; 2 Department of Sports Medicine Research Center of Sports Medicine Xiangya Hospital, Central South University, Hunnan, Changsha, 410008 China

**Keywords:** Posterior, Intervertebral space debridement, Bone graft, Fixation, Fusion

## Abstract

**Background:**

The choice of surgical methods for lumbosacral tuberculosis is controversial due to the complex anterior anatomy and peculiar biomechanics of the lumbosacral junction. The objective of this study was to explore the clinical effect of posterior intervertebral space debridement with annular bone graft fusion and fixation for the treatment of lumbosacral tuberculosis.

**Methods:**

We retrospectively analysed data from 23 patients with lumbosacral tuberculosis who had undergone posterior intervertebral space debridement with annular bone fusion and fixation between January 2008 and September 2014. The mean age of the patients was 49.0 years (range, 27–71), and the mean duration of disease until treatment was 10.2 months (range, 6–20). The lumbosacral angle, visual analogue scale (VAS) score, erythrocyte sedimentation rate (ESR), C-reactive protein (CRP) level, American Spinal Injury Association (ASIA) grade and Social Functioning-36 (SF-36) score were determined to ascertain the clinical effects of the treatment.

**Results:**

All patients underwent follow-up observation. The mean follow-up time was 34.2 months (range, 18–45), the mean operation time was 167.0 min (range, 130–210) and the mean blood loss was 767.4 ml (range, 500–1150). The lumbosacral angle was 21.0° ± 2.1° before operation, rising to 28.8° ± 1.7° after operation (*p* < 0.05) and being maintained thereafter. The mean VAS score before operation was 8.1 ± 0.6, decreasing to 1.2 ± 0.5 (*p* < 0.05) at the final follow-up. The mean ESR and CRP values were 49.1 ± 5.6 mm and 64.9 ± 11.9 mg/L, respectively, before operation, decreasing to normal at the final follow-up. The preoperative ASIA grade was C in 6 patients, D in 12 and E in 5. At the final follow-up, all patients had an ASIA grade of E except for one patient with a grade of D. For all patients, the SF-36 score at the final follow-up was higher than the preoperative and postoperative scores.

**Conclusions:**

Posterior intervertebral space debridement with annular bone graft fusion and fixation is an effective treatment for lumbosacral spine tuberculosis.

## Background

The lumbosacral region includes the distal lumbar vertebrae and all sacral vertebrae and mainly refers to L3 and lower levels [1, 2]. Tuberculosis of the lumbosacral region is rare, accounting for about 2%–3% of all cases of spinal tuberculosis [1, 3]. Although most cases of lumbosacral tuberculosis can be cured with conservative therapy, in some cases chronic low back pain and bad alignment of lumbar lordosis persist even with treatment. Surgical treatment is advocated for cases complicated by local instability or destructive vertebral body lesions.

Anterior debridement and bone grafting combined with posterior instrumentation is the leading surgical treatment for lumbosacral junction tuberculosis because it has the advantages of complete debridement under direct vision, excellent anterior column reconstruction and solid posterior fixation [3–5]. However, due to the complicated anatomic structure and unclear view of prevertebral inflammatory organisation, the risk of vascular, nerve and visceral injury is relatively high [6]. Moreover, accidental injury of the hypogastric nerve by this approach can bring about the severe complication of retrograde ejaculation in male patients [7, 8]. In consideration of these disadvantages, we tried the procedure of posterior intervertebral space debridement with annular bone graft fusion and fixation for the treatment of lumbosacral tuberculosis to ascertain the feasibility and effects of the procedure.

## Methods

### Patient population

This retrospective study enrolled 23 patients (12 men and 11 women) with lumbosacral tuberculosis who had undergone posterior transintervertebral space debridement with annular bone graft fusion and fixation between January 2008 and September 2014. The mean age of the patients was 49.0 years (range, 27–71), and the mean duration of disease until treatment was 10.2 months (range, 6–20). There were 3 patients with L3–4 lesions, 11 with L4–5 lesions and 9 with L5–S1 lesions. All patients were examined with anteroposterior and lateral X-rays. Computed tomography (CT) and magnetic resonance imaging (MRI) were performed to determine local pathological changes and eliminate other diseases of the lumbosacral region. All patients had lumbosacral pain; 14 patients had low fever, sweating, emaciation and other symptoms of tubercular toxicity; 17 patients had complications of radiating pain and numbness in the lower extremity; 18 patients experienced weakness in walking and 1 patient presented with cauda equina syndrome. On clinical examination, most patients presented only with lumbosacral pain, eight patients had limited lumbar flexion and six patients had significant weakness of the lower extremity. All preoperative erythrocyte sedimentation rate (ESR) and C-reactive protein (CRP) values were above normal limits. Except for seven patients who only presented with intervertebral space narrowing, all patients were found to have various degrees of bone destruction on X-ray examination. All patients were found to have obvious bone destruction and paravertebral abscesses on CT and MRI examination.

The study protocol was approved by the ethics review committee of the Shaanxi Provincial People’s Hospital (No. K20070313–021).

### Preoperative preparation

The T-SPOT.TB test was used to confirm the presence of tuberculosis. All patients with suspected tuberculosis received 2–4 weeks of standard HREZ chemotherapy (300 mg/day isoniazid, 450 mg/day rifampicin, 750 mg/day ethambutol and 750 mg/day pyrazinamide) before surgery. Anaemia and hypoproteinaemia were corrected preoperatively. If there was no significant decrease in the ESR, levofloxacin injection (i.v.gtt, 400 mg/d) was added to enhance the effect of the antituberculosis therapy.

### Operative technique

**Fig. 1:**
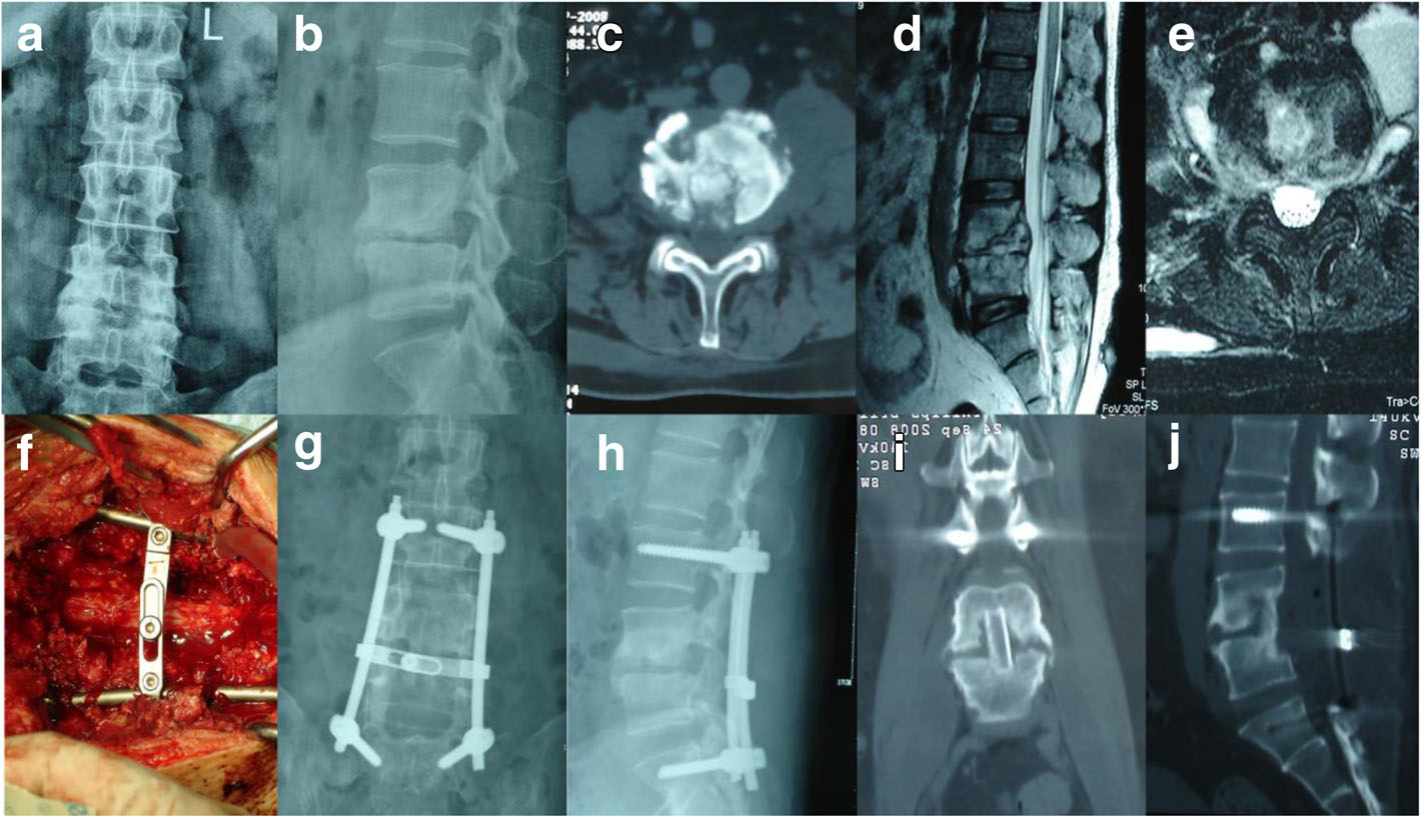
A 46-year-old female patient with lumbosacral tuberculosis with L4–5 lesions who underwent posterior intervertebral space debridement with annuar bone graft fusion and fixation. **a-e** Preoperative X-ray, computed tomography (CT) and magnetic resonance imaging showed bone destruction and abscess formation. **f** The intraoperative image showed posterior fixation, decompression and transintervertebral space debridement. **g-j** Postoperative radiographs. Postoperative CT in the axial and sagittal planes at 14 months showed a good position of internal fixation and excellent intervertebral bone fusion

**Fig. 2:**
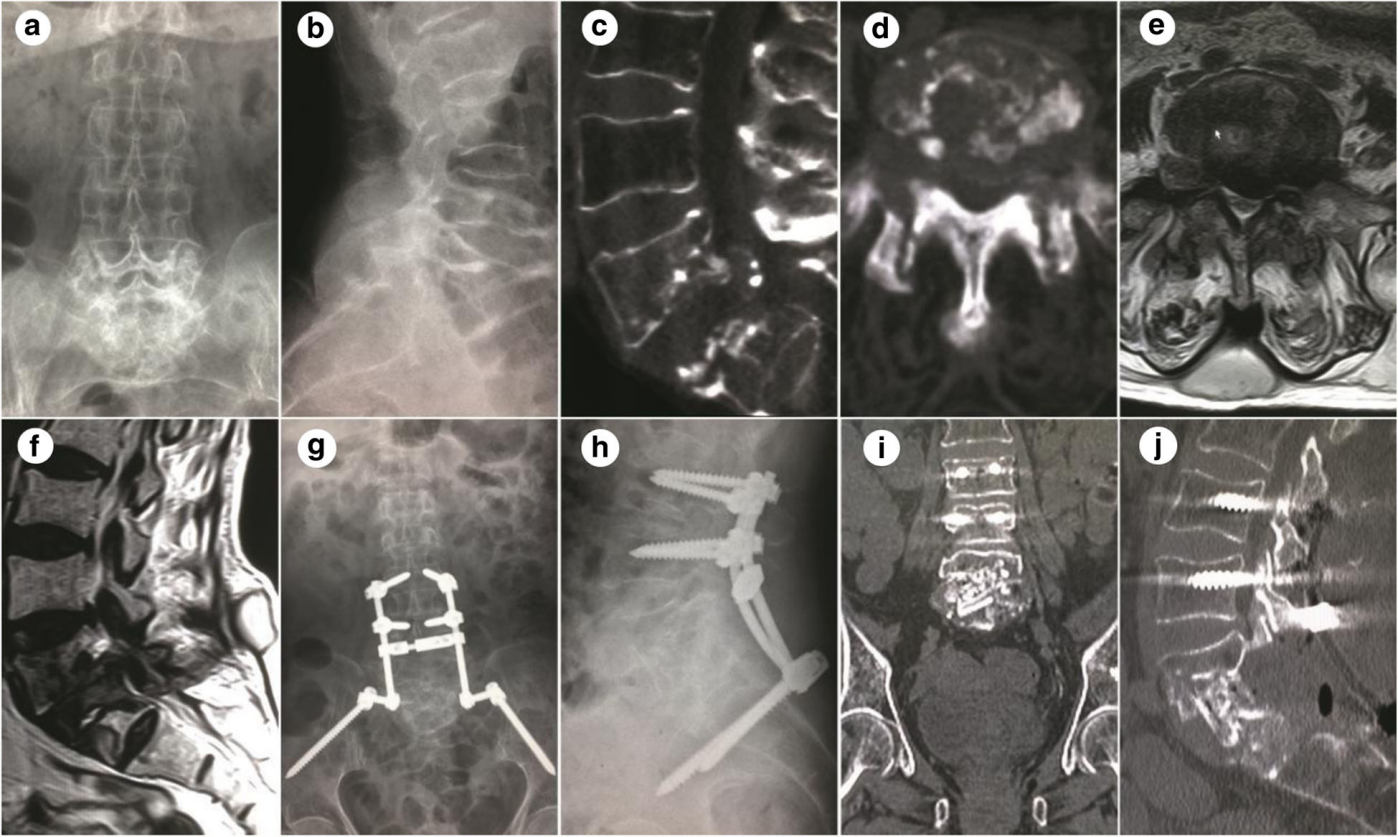
A 61-year-old female patient with lumbosacral tuberculosis with L5–S1 intervertebral lesion who also underwent posterior intervertebral space debridement with annular bone graft fusion and fixation. **a-f** Preoperative images. Preoperative computed tomography (CT) and magnetic resonance imaging showed bone destruction and extensive flow abscess (c–f). **g-j** Postoperative radiographs. Postoperative CT in the coronal and sagittal planes at 18 months showed a good position of internal fixation and excellent intervertebral bone fusion

With the patient in the prone position, a posterior midline skin incision was made after induction of general anaesthesia by tracheal intubation, and subperiosteal dissection was performed up to the lateral part of the superior zygopophysis. Pedicle screws were used in the adjacent normal vertebrae (Fig. 1). If the destruction of the pathological lumbar vertebrae was slight, pedicle screws were used. If the destruction of the S1 vertebra was severe, iliac screws were used for lumbopelvic fixation (Fig. 2). A short rod was temporarily installed to stabilise the operative segment during decompression and focal debridement. Total laminectomy under protection of the facet joints was performed, and cotton pieces were used to protect the dura. Under protection of the dura and nerve roots, curettes of various sizes and angles and high-speed burrs were used to remove abnormal bone (especially sclerous bone) down to healthy haemorrhagic bone. A 90° curette was used cautiously to push and scrape paravertebral pathological tissue, and a positive pressure catheter was inserted into the intervertebral space or paravertebral tissue to drain pus and assist in clearing necrotic tissue. We trimmed the bone grafting area and extracted the bone fragment from the iliac (iliac block) and granules, then put the iliac block into the intervertebral space and tamped osseous granules around the iliac block. We cut the lamina into pieces and implanted them around the facets and the transverse processes. The diseased material was sent for microorganism culture and histopathological examination.

### Postoperative care

Breath, pulse, blood pressure and temperature were monitored postoperatively. Mobility and sensation of the lower limbs were closely observed. The drainage tube was extracted when the volume of drainage was <50 ml/d. The patients underwent straight leg raising exercises and ankle pump training during bed rest. We regularly monitored routine blood parameters, ESR, CRP and liver function, performed X-rays of the lumbar spine and, if necessary, performed CT scanning of the surgical segment. Standard HREZ chemotherapy was continued for more than 12 months. Ambulation with a brace was allowed at 6–8 weeks after surgery. The patients performed non–weight-bearing daily activities until radiographic (X-ray or CT) evidence of fusion was observed. The patients then returned to normal weight-bearing activities.

### Postoperative evaluation

Preoperatively and at the final follow-up, the American Spinal Injury Association (ASIA) grade was used to evaluate neurological function. At the same time points, the visual analogue scale (VAS) was used to assess low back pain, and tuberculosis activity was monitored by measuring ESR and CRP. The lumbosacral angle and Social Functioning-36 (SF-36) scores were measured at preoperation, postoperation and final follow-up. Bone graft fusion and instrumentation failure were monitored by radiography.

### Statistical analysis

The paired *t-*test was used to evaluate differences in lumbosacral angle, VAS, ESR, CRP and SF-36 among preoperation, postoperation and final follow-up. The Wilcoxon signed rank test was used to evaluate differences in ASIA grade. A *p*-value <0.05 was considered to indicate a statistically significant difference.

## Results

All patients underwent follow-up observation. The mean follow-up time was 34.2 months (range, 18–45), the mean operation time was 167.0 min (range, 130–210) and the mean blood loss was 767.4 ml (range, 500–1150) (Table 1). Except for one relapsed patient who was cured by debridement of a second abscess via a small anterior incision, all patients achieved stage 1 healing. The lumbosacral angle was 21.0° ± 2.1° before operation, rising to 28.8° ± 1.7° after operation (*p* < 0.05) and being maintained thereafter. The difference between the lumbosacral angle before and after operation was statistically significant (*p* < 0.05), and there was no significant loss of postoperative lumbosacral angle during follow-up (*p* > 0.05). Tuberculosis toxic symptoms had vanished in all patients at the final follow-up. The mean VAS score at the final follow-up was 1.2 ± 0.5 (range, 0–3), significantly lower than the mean preoperative score (8.1 ± 0.6) (Table 2).

**Table 1:** Patient demographics, operative information and disease characteristics

Case no.	Age (years)/sex	Level	Duration of symptoms (months)	Blood loss (ml)	Operation time (min)	Follow-up (months)
1	49/M	L5-S1	13	1150	200	45
2	51/F	L4–5	8	1000	150	38
3	49/M	L5-S1	12	700	150	35
4	48/F	L4–5	9	950	160	30
5	57/M	L5-S1	11	1050	210	38
6	45/F	L5-S1	7	600	140	31
7	45/M	L4–5	14	900	170	28
8	51/M	L3–4	7	850	150	31
9	71/M	L5-S1	6	500	170	38
10	48/F	L5-S1	8	750	210	36
11	63/F	L3–4	9	800	160	45
12	51/M	L5-S1	20	800	200	18
13	37/M	L4–5	9	650	190	36
14	42/M	L4–5	13	800	170	42
15	61/F	L5-S1	8	950	200	28
16	47/F	L4–5	13	500	130	35
17	55/F	L4–5	10	550	140	31
18	35/M	L4–5	9	750	180	42
19	46/F	L4–5	11	600	130	29
20	52/F	L4–5	9	850	170	32
21	55/M	L5-S1	10	500	140	36
22	41/F	L3–4	12	700	150	35
23	27/M	L4–5	6	750	170	28
Mean	49.0 ± 9.5		10.2 ± 3.2	767.4 ± 181.2	167.0 ± 25.1	34.2 ± 6.3

**Table 2:** Summary of radiological and clinical outcomes

Case no.	Lumbosacral angle (°)			ASIA grade		VAS		ESR (mm/h)		CRP (mg/L)	
	Pre	Post^*^	Final	Pre	Final^Δ^	Pre	Final^*^	Pre	Final^*^	Pre	Final^*^
1	19	28	27	C	E	8	1	46	15	69	3.0
2	25	31	30	C	E	7	0	42	13	48	1.5
3	25	30	28	D	E	9	1	39	12	45	2.5
4	22	29	27	E	E	9	1	48	13	51	3.5
5	18	27	26	E	E	8	1	52	10	79	2.0
6	21	27	27	E	E	7	1	49	13	71	2.5
7	19	27	25	C	E	9	0	52	11	83	3.5
8	20	26	25	D	E	9	1	65	12	97	3.0
9	26	33	32	E	E	8	2	58	14	85	3.5
10	20	29	29	E	E	8	1	61	13	91	1.0
11	25	32	31	C	D	8	1	37	14	50	4.5
12	18	25	23	C	E	7	2	38	15	48	3.5
13	17	27	26	D	E	8	2	47	12	55	3.5
14	24	30	30	D	E	7	0	45	13	56	3.0
15	19	29	28	D	E	9	2	57	10	69	2.5
16	22	29	27	D	E	9	2	53	14	75	1.5
17	21	28	28	D	E	8	1	50	11	64	2.0
18	19	27	26	D	E	8	3	47	13	63	2.5
19	21	30	29	D	E	7	1	56	13	68	2.5
20	18	27	27	C	E	8	1	39	11	47	2.0
21	23	32	30	D	E	9	1	49	12	52	3.5
22	21	31	28	D	E	8	1	48	14	62	3.5
23	20	29	29	D	E	8	1	51	13	65	3.0
Mean	21.0 ± 2.1	28.8 ± 1.7	27.7 ± 1.7			8.1 ± 0.6	1.2 ± 0.5	49.1 ± 5.6	12.7 ± 1.1	64.9 ± 11.9	2.8 ± 0.7

*Pre* preoperative, *Post* postoperative, *Final* final follow-up, *ASIA* American Spinal Injury Association, *VAS* visual analogue scale, *ESR* erythrocyte sedimentation rate, *CRP* C-reactive protein

^*^ *p* < 0.05 compared with preop by paired *t*-test

^Δ^ *p* < 0.05 compared with preop by Wilcoxon signed rank test

ESR and CRP were recovered to normal levels (12.7 ± 1.1 mm/h and 2.8 ± 0.7 mg/L, respectively) at the final follow-up. At the final follow-up, all patients had an ASIA grade of E except for one patient with a grade of D (Table 2). The postoperative SF-36 scores for Physical Functioning, Role-Physical, Bodily Pain, General Health, Vitality, Social Functioning, Role-Emotional and Mental Health were 80.94 ± 3.30, 29.70 ± 14.24, 69.5 ± 13.06, 70.27 ± 9.92, 78.37 ± 9.86, 73.08 ± 9.88, 63.43 ± 18.70 and 83.78 ± 2.97, respectively. All these values were significantly (*p* < 0.05) higher than the preoperative scores of 77.43 ± 4.67, 27.0 ± 17.05, 24.59 ± 0.50, 68.2 ± 11.25, 72.43 ± 9.83, 69.00 ± 6.26, 59.8 ± 13.77 and 81.40 ± 3.55. The scores for each part of the SF-36 increased gradually during the follow-up period, and all scores were significantly (*p* < 0.05) higher than the postoperative scores at the final follow-up (Table 3). All grafted bone achieved solid fusion according to X-ray examination (partial fusion by CT scan); the mean time to bone fusion was 6.5 months (range, 6–10). There was no loose fixation, dislocation or rupture.

**Table 3:** Preoperative, postoperative and final follow-up SF-36 scores

Characteristic	Preop	Postop	Final follow up
PF	77.43 ± 4.67	80.94 ± 3.30^*^	87.43 ± 8.38^Δ^
RP	27.0 ± 17.05	29.70 ± 14.24^*^	39.8 ± 12.44^Δ^
BP	24.59 ± 0.50	69.5 ± 13.06^*^	78.57 ± 3.56^Δ^
GH	68.2 ± 11.25	70.27 ± 9.92^*^	77.70 ± 7.87^Δ^
VT	72.43 ± 9.83	78.37 ± 9.86^*^	85.90 ± 11.42^Δ^
SF	69.00 ± 6.26	73.08 ± 9.88^*^	81.10 ± 13.01^Δ^
RE	59.8 ± 13.77	63.43 ± 18.70^*^	72.50 ± 20.52^Δ^
MH	81.40 ± 3.55	83.78 ± 2.97^*^	89.08 ± 6.64^Δ^

*SF-36*, Social Functioning-36, *PF* Physical Functioning, *RP* Role-Physical, *BP* Bodily Pain, *GH* General Health, *VT* Vitality, *SF* Social Functioning, *RE* Role-Emotional, *MH* Mental Health, *preop* preoperation, *postop* postoperation

^*^ *p* < 0.05 compared with preop by paired *t*-test

^Δ^ *p* < 0.05 compared with postop by paired *t*-test

## Discussion

With the resurgence of tuberculosis, the incidence of spinal tuberculosis has increased in recent years [9, 10]. Lumbosacral junction tuberculosis accounts for only a small proportion of spinal tuberculosis (about 2%–3%) [3, 11], but due to the anatomical and biomechanical particularities of the lumbosacral junction, its treatment is controversial. The clinical manifestation of lumbosacral junction tuberculosis is slightly different from that of typical thoracic and lumbar tuberculosis, usually presenting only as slight low back pain, occasionally accompanied by symptoms of sciatic nerve irritation. There is never severe neurological dysfunction or kyphosis [12].

The blood supply of the lumbosacral junction is poor. Once *Mycobacterium tuberculosis* has migrated to and caused disease in this site [13]. Moreover, the lumbosacral junction is one of the most significant stress-concentrating segments of the spine (a transitional segment from an active lumbar vertebra to a fixed sacral vertebra) and is hard to immobilise and rest. As a result, the lumbosacral junction is difficult to treat [14].

The application of antituberculosis drugs is the basis of the treatment of spinal tuberculosis [15]. In the early stage of spinal tuberculosis, antituberculosis drugs can effectively reach the lesion area, but with the progress of the disease, the local blood flow of the lesion gradually deteriorates, while the tuberculosis bacilli are wrapped in granulation and necrosis, so that the drugs cannot exert an effective antibiotic action against the tuberculosis bacilli that are residing in the lesion [16]. Appropriate surgical therapy can significantly improve the local blood supply, break the drug barrier, shorten the course of the disease and reduce the occurrence of complications [17].

The aim of surgical treatment of lumbosacral junction tuberculosis is radical debridement to wipe out the factors detrimental to neural recovery and restore normal spine alignment. An anterior approach can directly remove paravertebral abscesses and vertebral sequestrum and perform effective intervertebral grafting, but due to the complex anterior anatomy of the lumbosacral junction, its ambiguous pathological structure, fragile invading blood vessels and thick, sticky adjacent tissue, the risk to the presacral vessels and the ureter is increased [18]. Accidental injury to the hypogastric nerve in male patients may lead to the severe complication of retrograde ejaculation [7, 8]. Moreover, the backward slant of the sacrococcyx makes implantation in the anterior region difficult [19]. Even if fixation in the anterior region is performed during anterior surgery, the instrument is difficult to extract later because of the complicated anterior anatomic structures. Considering these difficulties, some surgeons have adopted combined anterior and posterior surgery to treat patients with lumbosacral junction tuberculosis. The normal sequence of the lumbosacral junction can be established through solid posterior fixation. Moreover, by effective anterior focus clearance and intervertebral bone grafting, the stability of the lumbosacral junction can be maintained in the long run [2, 20]. Furthermore, the lumbosacral junction needs no extensive anterior exposure. However, the surgical trauma is great, and the operative position needs to be changed [21].

Pang et al. [22] and Zhang et al. [23] reported that the procedure of one-stage posterior transforaminal debridement, interbody fusion and instrumentation achieved a good result for lumbosacral junction tuberculosis. In our study, we performed debridement via the intervertebral space by taking the paramedial approach rather than the transforaminal approach, because we have experience with this approach and we can reach the intervertebral space easily on both sides, remove debris, insert the graft and correct the deformity. In this study, only one patient relapsed. We believe the success of debridement by this procedure was due to our strict selection of patients and our proficient posterior spinal skills. This procedure is not suitable for patients with extensive paravertebral abscesses or flow abscesses.

The following are some notes on this technique. First, there is no problem of dura avulsion [24]. Due to the narrow space of debridement, the risk of dura laceration is theoretically higher. In our study, there were six cases of dura laceration. All patients were healed by fluid infusion and relevant management (the dura was sutured in three patients), and there was no spread of infection or secondary intracranial infection. We suggest that if dura tear occurs it should be sutured if possible during the operation. If the dura cannot be sutured, the deep fascia must be rigid sutured. Remember to remove the drainage tube as quickly as possible and suture the drainage entrance deeply. Second, decrease the traction on the neural root. When we remove the pathologic sclerotic bone and the paravertebral abscess, it is hard to avoid traction and irritation of the neural root. Our experience is that preoperative preparation with spatulas of various angles, osteotomes and high-speed drills is essential for this operation. During the operation, the surgeon needs to be gentle. If the lesion is difficult to deal with ipsilaterally, it can be approached on the contralateral side in the intervertebral space. Third, protect the anterior longitudinal ligament when clearing the paravertebral abscess. If the abscess is too viscous, high-pressure washing should be used [25]. Rough surgical techniques on the anterior side of the vertebrae with no visual confirmation are forbidden. Fourth, use the combined structural and granulated bone impaction grafting method for intervertebral bone grafting to decrease the risk of neural injury and increase the efficacy of bone fusion [26].

## Conclusions

Transintervertebral space debridement with annular bone graft fusion and fixation through a single posterior approach is a good treatment for lumbosacral junction tuberculosis because of its effective debridement, bone grafting and fixation.

## Abbreviations

ASIA: American Spinal Injury Association
CRP: C-reactive protein
CT: Computed tomography
ESR: Erythrocyte sedimentation rate
Final: Final follow up
MRI: Magnetic resonance imaging
SF-36: Social Functioning-36
VAS: Visual analog scale
